# Perspective: Nutrition Research & Programming in Multicultural Populations: The Fixed-Quality Variable-Type (FQVT) Dietary Intervention^☆^

**DOI:** 10.1016/j.advnut.2025.100505

**Published:** 2025-09-04

**Authors:** David L Katz, Christopher D Gardner

**Affiliations:** 1 Diet ID, Detroit, MI, United States; 2 True Health Initiative, The Health Sciences Academy, London, United Kingdom; 3 Prevention Research Center, Stanford University School of Medicine, Stanford, CA, United States

**Keywords:** nutrition research, dietary assessment, methodology, diet quality, personalized nutrition, multiculturalism

## Abstract

The sine qua non of intervention studies in general, and randomized controlled trials in particular, is to define and isolate an exposure of interest that defines the intervention and distinguishes between groups. The isolation of a presumptive cause is a prerequisite to the confident attribution of given effects. In the context of dietary intervention studies, this has historically translated into a unitary intervention diet type, no matter the diversity of preferences, tastes, upbringings, ethnicities, and cultures represented in a given study cohort. To the extent such diversities have been constrained to achieve this aim, generalizability (external validity) has been diminished. To the extent such diversities have been ignored, inattention to them has likely shifted results toward the null and compromised adherence over time. These same liabilities pertain to food service projects and public health nutrition, notably the food-as-medicine movement. We propose a remedy to these issues and an update to the formula for dietary intervention research (and service) that accommodates a multicultural society: the fixed-quality, variable-type (FQVT) nutrition intervention. This method standardizes the objective measure of diet quality and incorporates fixed tolerances for nutrients of particular interest, while allowing for a range of diet types responsive to the variable preferences of study participants/population members. We describe the application of tools to facilitate this methodological innovation, enumerate the expected advantages, and characterize means of empirical testing. We submit the FQVT method as a promising, testable advance in the evolution of clinical nutrition research and food-is/as-medicine programming.

**Statement of significance**

This article introduces the fixed-quality, variable-type (FQVT) construct for dietary intervention research and programming. The particular novelty of the FQVT approach resides in the opportunity to include in a single study or program any given plurality (i.e., not merely 2 or several) of dietary patterns, personalized at the n-of-1 level, yet standardized to within a prespecified tier of objectively measured diet quality, and matched in addition for nutrient ranges of relevance and interest.

## Introduction

The sine qua non of intervention studies in general, and randomized controlled trials (RCTs) in particular, is to define and isolate a single exposure of interest that defines the intervention and distinguishes between groups [1]. The isolation of a presumptive cause is a prerequisite to the confident attribution of given effects. Because diet is an intrinsically complex variable; because dietary intake cannot be “blinded”; and because a “placebo diet” does not exist—the adaptation of methods developed preferentially for drug trials to nutrition research has evolved into a realm of often contentious debate [2,3]. Presumably to approximate the features of RCTs in other domains, dietary intervention studies focused on overall dietary patterns have historically offered (imposed?) a single diet type, no matter the diversity of preferences, tastes, upbringings, ethnicities, and cultures represented in a given study cohort. The same practice now tends to prevail in service programs, such as cardiac rehabilitation and diabetes prevention [4]. Medically tailored meals in the food-is/as-medicine context may similarly neglect the importance of multicultural variants [5]. Because clinical and public health programming (e.g., diabetes prevention, cardiac rehabilitation, WIC) are informed by research evidence, inattention to multicultural dietary variation in the research domain reverberates to the service domain. Historical inattention to multicultural considerations in dietary guidance has been the subject of recent criticism [6].

Taste is so prototypically personal that it serves as a metaphor for personal preference of every variety (e.g., “a matter of taste;” in French, “a chacun son gout”). Yet, this universal reality is routinely ignored in dietary intervention research. The standard approach in dietary intervention studies focused on overall diet, whether predicated on guidance, the direct provision of food, or both, is to define the intervention by dietary pattern [e.g., the dietary approaches to stop hypertension (DASH) diet, a Mediterranean diet, etc.].

An emphasis on diet type rather than diet quality has in general propagated fissiparousness and misguided allegiances (so-called “diet tribes”); obscured fundamentals of healthful eating that transcend any fixed typology (e.g., a generous intake of vegetables); diverted both public and professional attention to such trivial, type-based distractions as macronutrient thresholds [7,8]; and Balkanized nutrition research. Researchers working in the “silo” of a given diet type (e.g., the Mediterranean diet) could produce results that are contrasted to those of researchers studying an alternative type (e.g., a whole food, plant-based diet) [9], with potential inattention to the more important commonalities [10]. A siloed and competitive approach in research is reinforced when translated to clinicians and the public at large by both scholarly journals and the media, which share and amplify type-based arguments. When competing diet types have been directly compared for specific outcomes, from weight loss to cardiometabolic health, the results consistently highlight the importance of fundamental nutrition features (e.g., energy restriction if weight loss is the goal; an emphasis on vegetables, fruits, legumes, nuts and seeds if cardiometabolic health is the goal) over type-based differentiation [[11], [12], [13]].

The importance of accommodating individual preference and cultural diversity has emerged as a recent theme in nutrition commentary and practice guidelines [14], including the recent work of the 2025 Dietary Guidelines Advisory Committee [15]. Historical inattention to this imperative has garnered rebuke [6]. The United States is home to a global assembly of hundreds of ethnicities/cultures, with many dozens of such population subgroups numbering in the hundreds of thousands to millions [16]. Among Asian cultures alone, ≥6 are represented in the United States by populations well over a million [17]. Of note, most natives of East Asia are genetically lactose intolerant [18] (the normal condition among adult mammals of all species, with the exception of selectively adapted Homo sapiens) and most diets of East Asia traditionally exclude dairy altogether. Dairy, however, is a mainstay in leading “intervention” diets used in research studies, notably the DASH diet [19] and the dietary pattern in the Diabetes Prevention Program (DPP) [20], and is of variable salience in the Mediterranean diet and the Mediterranean-DASH intervention for neurodegenerative delay diet [21].

Diet quality measured objectively—not a choice among dietary patterns of comparable quality—has been identified as the single leading predictor of premature death and chronic disease in the United States and much of the developed world [22,23]. Methods have long existed in principle to “fix” diet quality across a range of diet types; diverse menu plans could be developed to hit prespecified nutrient and quality targets [24]. Recent innovations provide means to do so far more practically and efficiently, to adapt a fixed diet quality across a range of cultural variants [25], and to contrast intervention diet quality to individual baseline at scale [26,27]. To our knowledge, there has thus far been no translation of the evolving mandate to accommodate multiculturalism into the structure of dietary intervention research or standardized nutrition intervention programs (e.g., the DPP, cardiac rehabilitation [28,29]). In this article, we introduce the fixed-quality, variable-type (FQVT) dietary intervention study, enumerate the salient methodologic features, characterize tools to facilitate the approach, and explore potential implications.

## Approach

A FQVT nutrition study begins with the establishment of the requisite diet quality and its objective quantification. This is achieved using a validated measure, such as the Healthy Eating Index (HEI) 2020 [24], and defining a range of values within which to standardize the quality of all intervention diets. That range could be wider (e.g., within a quintile) or narrower (e.g., within a decile) as per the demands of any given study.

The HEI, among the most widely used and robustly validated of diet quality measures, is populated with both food-level and nutrient-level components. Accordingly, fixing diet quality by this measure, and in particular fixing it at or near the top of the 100-point scale, intrinsically fixes the proportional distributions of food groups and nutrients within ranges compatible with the diet score [30]. Further stipulations regarding foods or nutrients could be imposed in accordance with a study hypothesis: fiber (total, soluble, insoluble, or each separately) could be set at an especially high level; sodium could be set particularly low; and so on. Such nutrient tolerances, like overall diet quality, can readily be extended across a range of diet types [31]. Food groups can similarly be delivered in targeted ranges, such as some daily mean for vegetable or legume servings in proportion to calories (e.g., X daily servings of vegetables/1000 kcal).

The above steps anticipate that a wide range of culturally tailored diets—accounting for social context, ethnicity, upbringing, and family traditions—can be standardized to the same, high-quality range. This has been established empirically with the use of the HEI-2020 [26] and is supported by real-world outcomes as well [32,33]. Because some time-honored ethnic diets, including ≥1 associated with noteworthy longevity and health span [[34], [35], [36]], exclude certain food groups, notably dairy, this approach invites an adaptation of HEI-based diet quality scoring to allow for exclusion of “discretionary” (i.e., not universally distributed across populations; not established to be an essential component of dietary patterns producing optimal health outcomes) food groups. Dairy is routinely excluded from East Asian diets, and epitomizes the importance of this accommodation so that the absence of a discretionary food group does not attenuate the “credit” a given dietary pattern can earn. The omission of grains from some versions of a Paleolithic diet is another example. An approach to just such adaptations of the HEI for multicultural applications has recently been introduced [25]. Thus, a wide range of diets with variance in foods, and to a lesser extent, food groups, could be standardized to the same narrow range of diet quality, and any nutrient ranges of special relevance to the intervention program or study.

The establishment of a range of dietary patterns meeting prespecified quality and nutrient criteria, and spanning the ethnicities (or at least as many of them as practicable) of study participants/population members then allows for the presentation to each individual some plurality of dietary patterns from which to choose, catering to demographic profile and variations in taste and preference, while honoring the study hypothesis, research protocol, and/or intended outcomes. Participants may be guided to make a selection by any established means [37], including a recent innovation that displays the options in images [38,39]. This approach highlights the importance of expertise in implementation science in protocol development and execution [40].

Intervention components—whether limited to guidance for dietary behavior change, or extending to the provision of food—could then be matched to the range of dietary patterns selected, as well as the prespecified nutrition criteria. Resources would need to be available to develop and deliver both personalized guidance and dishes to accommodate the approach. Such models exist [41]

The specific sequence of methods aligned with this construct is as follows:

1) Conduct a comprehensive assessment of dietary intake and overall diet quality at baseline/enrollment.
2) Fix the objective quality (e.g., as measured by the HEI 2020) standard for intervention dietary patterns within a desired range, and establish tolerances for specific nutrients of interest.
3) Offer a plurality of dietary patterns responsive to specific outcome objectives for any given trial, including high-quality versions of culturally tailored diets, and invite individuals to make a favored selection.
4) Administer the dietary intervention by any means consistent with the standards of practice: provide general guidance; provide a detailed meal plan and guidance; directly provide some food/meals; directly provide all food/meals; provide and measure all food/meals on a metabolic ward. Match guidance and dishes to prespecified nutritional criteria.
5) Reassess dietary intake and overall diet quality at suitable intervals during the trial.
6) Analyze the effects of the intervention on prespecified outcomes using the following as independent variables: the intended diet quality and nutrient levels; the as-measured diet quality and nutrient levels achieved; and the as-measured change from baseline in diet quality and nutrient levels of interest.

## Discussion

Tastes and preferences vary. Familiarity is among the more potent determinants of dietary preference [42], and it, in turn, varies with ethnicity, upbringing, social context, family traditions, and even pre and perinatal exposures. Among important limitations to the burgeoning food-is/as-medicine movement is the paucity of data demonstrating successful, long-term adherence to prescribed improvements in dietary intake [41]. The extent to which adherence is compromised by one-size-fits-all dietary interventions that ignore culture and personal preference is uncertain, but both qualitative research [43] and informed intuition suggest that the role is likely considerable.

Large, prospective cohort studies suggest that improvements in health track consistently and robustly with improvements in objectively measured overall diet quality, without regard to specified diet type [[44], [45], [46], [47], [48]]. This contention is supported by the Blue Zones ethnography, which demonstrates the same, enviable health and longevity outcomes across a diversity of culturally distinct diet types consistent in fundamental nutrition themes [49,50] and overall quality. The observational indications that diet quality, not type, is the determinant of pertinent outcomes invite both the testing of this hypothesis in controlled intervention trials and the accommodation of individual preference in the service of enhanced outcomes and lasting adherence in all food-is/as-medicine programming.

The methods enumerated above are manageable with tools long available, such as semiquantitative food frequency questionnaires or multiday food journals, and their modern adaptations to digital environments [51,52]. They are much facilitated by newly available, validated methods [38,53] that can compress dietary assessment from days and hours to minutes and seconds, and deliver an objective diet quality score instantaneously. Recent innovation also allows for simple adaptations of established measures of diet quality to accommodate variations in food group inclusions across a multicultural expanse [25].

The particular novelty of the FQVT approach resides in the opportunity to include in a single study or program any given plurality (i.e., not merely 2 or several) of dietary patterns, personalized at the n-of-1 level, yet standardized to within a prespecified tier of objectively measured diet quality, and matched in addition for nutrient ranges of relevance and interest. In scanning the published literature for the combination of “randomized trial” and “personalized nutrition,” there are relatively few articles overall, with nearly all predicated on biologically (e.g., genomics, metabolomics) based personalization [[54], [55], [56], [57]]. Furthermore, such studies tend generally to accommodate 2 or, at most, several dietary patterns, not true personalization (i.e., at the n-of-1 level). Thus, personalization is group based, or predicated on a predetermined dietary comparison (e.g., low carb compared with low fat) as has been done both without [7] and with [58] genotype-based assignment. Such articles indicated that the tailoring of meals is generally for a given population, rather than allowing for individualized variation based on such factors as upbringing, cultural heritage, and personal preference—with or without biological (e.g., genomic, metabolomic, etc.) differentiation.

Personalization based on such factors as cultural heritage (e.g., allowing a diverse group of study participants from different cultural backgrounds to select a dietary pattern native to their upbringing) and personal preference is generally not mentioned, nor is there mention in this literature of standardizing across a plurality of diet styles within a narrow, prespecified tier of objectively measured diet quality (i.e., the HEI-2020) along with a suite of nutrient-specific tolerant ranges (e.g., sodium, added sugar, fiber, calcium, and omega-3 fat) germane to the study questions. As noted previously, the FQVT approach is novel in all of these particulars.

Dietary intervention research and service programs in public health nutrition, including food-is/as-medicine programming, could readily emulate this example. The functional objectives residing in the distribution of food groups, nutrient ranges, and diet quality could be standardized and retained, whereas the form represented by diet type is allowed to vary. The potential advantages encompass, but are not limited to: enhanced participant satisfaction, enhanced adherence, maintenance of dietary practices over time, and the preservation of all key determinants of internal validity, with greatly augmented external validity.

### Limitations

Any innovation in methods is apt to involve some trade-offs, and the FQVT intervention design is no exception. If providing food, then tailoring to a wide range of personal preferences requires greater flexibility and inventory in the source kitchen, and higher-order orchestration to ensure the right food is delivered to each participant. This is apt to be challenging, if not daunting, for researchers and public health practitioners alike. Expertise in implementation science is apt to be helpful. In addition, new means of sourcing multicultural menu plans are among the innovations spawned by the food-is/as-medicine movement [41]. Researchers and health departments alike could potentially outsource this challenge to private sector partners (for profit or nonprofit) with pertinent resources, expertise, and supply chains.

If providing dietary guidance without direct provision of food, the tailoring of this to the n-of-1 level to align with personal dietary selection similarly increases logistical demands. These challenges would vary quantitatively with the size and diversity of the study cohort or service population. These same demands, however, must be addressed to accommodate real-world populations, and thus meeting them in the context of research is a bow to verisimilitude. Moreover, digital innovations allow for the delivery of guidance curated to a personalized goal diet, and the burgeoning food-is/as-medicine ecosystem is a resource for meeting the culinary demands of a diverse population. Applicable here as well are the advances associated with the use of artificial intelligence, including facilitation of personalized goal diet selection [37] and the automated personalization of coaching to that goal diet [59]. Adaptations of specific dietary objectives could readily contextualize them in dietary patterns responsive to family traditions, upbringing, social context, cultural imperatives, and personal preferences, with corresponding guidance and counseling.

The FQVT approach is conducive to testing in the context of randomized, controlled trials. The intervention may be directed to any given primary outcome of interest (e.g., improvement in HgbA1c levels), and a suitable control condition established. That condition could be a waiting list, with delayed intervention; a passive control condition of “usual care;” or the assignment of a traditional “one-size-fits-all” intervention diet. A 3-arm study, in which 1 active intervention group receives variable-type, and another receives fixed type diets, matched for quality, with both compared with a nonintervention control, is of potential interest. Although by no means the only pertinent design, this construct would allow for determination of how much impact each approach had on diet quality and related objectives, allowing for determination of cost-effectiveness and return on investment in such programming [60].

Tools noted above allow for the rapid, efficient, and reliable establishment of baseline diet quality, and the tracking of changes in diet quality over the study span and after. Generating a plurality of culturally/personally adapted diets matched for quality, applying a common standard of diet quality fairly across such an expanse, and presenting a curated set of dietary choices to each study participant are all enabled by extant tools and recent innovations.

In conclusion, a former senior official at the Centers for Disease Control and Prevention (CDC), Prof Larry Green, coined the aphorism that if we “…want more evidence-based practice, we need more practice-based evidence.” The practice of dietary intake by everyone, on a daily basis, is characterized by personal preference, personal choice, and variation in taste. The FQVT approach to research (and public health nutrition programming) preserves the essential elements of internally valid dietary intervention research, while accommodating this real-world imperative in the service of satisfaction, adherence, maintenance, social equity, and external validity. Empirical testing is warranted.

## Author contributions

The authors’ responsibilities were as follows – DLK: responsible for the conceptualization of FQVT and for drafting the initial version of the manuscript; and all authors: participated in refining the concept and its implications, and in editing of the manuscript and read and approved the manuscript for submission/publication.

## Funding

The authors reported no funding received for this study.

## Conflict of interest

DLK reports a relationship with Diet ID Inc that includes: employment and equity or stocks. DLK has a patent #United States Patent No. 11,328,810 B2 issued to Diet ID Inc. DLK has patent #United States Patent No. 12,073,935 B2 issued to Diet ID Inc. DLK has a patent Dietary Impacts on Environmental Measures (DIEM), pending to Diet ID Inc. DLK has patent Adaptive Component Scoring for Diet Quality Assessment pending to Diet ID Inc. All other authors report no conflicts of interest.
